# The Parkinson’s disease-associated GPR37 receptor interacts with striatal adenosine A_2A_ receptor controlling its cell surface expression and function *in vivo*

**DOI:** 10.1038/s41598-017-10147-x

**Published:** 2017-08-25

**Authors:** Xavier Morató, Rafael Luján, Marc López-Cano, Jorge Gandía, Igor Stagljar, Masahiko Watanabe, Rodrigo A. Cunha, Víctor Fernández-Dueñas, Francisco Ciruela

**Affiliations:** 1 Unitat de Farmacologia, Departament Patologia i Terapéutica Experimental, Facultat de Medicina, IDIBELL, Universitat de Barcelona, L’Hospitalet de Llobregat, Barcelona, Spain; 2 Institut de Neurociències, Universitat de Barcelona, Barcelona, Spain; 3 IDINE, Departamento de Ciencias Médicas, Facultad de Medicina, Universidad Castilla-La Mancha, Albacete, Spain; 4 Donnelly Centre, Department of Molecular Genetics, Department of Biochemistry, University of Toronto, Toronto, M5S 3E1 Canada; 5 Department of Anatomy, Hokkaido University School of Medicine, Sapporo, 060-0818 Japan; 6 CNC-Center for Neuroscience and Cell Biology, Faculty of Medicine, University of Coimbra, Coimbra, Portugal

## Abstract

G protein-coupled receptor 37 (GPR37) is an orphan receptor associated to Parkinson’s disease (PD) neuropathology. Here, we identified GPR37 as an inhibitor of adenosine A_2A_ receptor (A_2A_R) cell surface expression and function *in vivo*. In addition, we showed that GPR37 and A_2A_R do oligomerize in the striatum. Thus, a close proximity of GPR37 and A_2A_R at the postsynaptic level of striatal synapses was observed by double-labelling post-embedding immunogold detection. Indeed, the direct receptor-receptor interaction was further substantiated by proximity ligation *in situ* assay. Interestingly, GPR37 deletion promoted striatal A_2A_R cell surface expression that correlated well with an increased A_2A_R agonist-mediated cAMP accumulation, both in primary striatal neurons and nerve terminals. Furthermore, GPR37−/− mice showed enhanced A_2A_R agonist-induced catalepsy and an increased response to A_2A_R antagonist-mediated locomotor activity. Overall, these results revealed a key role for GPR37 controlling A_2A_R biology in the striatum, which may be relevant for PD management.

## Introduction

GPR37, also known as parkin-associated endothelin-like receptor (Pael-R), is an orphan G protein-coupled receptor (GPCR) expressed in brain regions such as cerebellum, corpus callosum, caudate nucleus, putamen, substantia nigra and hippocampus^1, 2^. The physiological function of this receptor has not been still elucidated. Thus, GPR37 is known to be expressed in neural progenitor cells and control Wnt signaling^3^. Also, a GPR37-mediated control of oligodendrocyte differentiation has been recently described^4^, but scarce information exists regarding its presence in other neuronal subsets. On the other hand, the neuropathological role of GPR37 has been extensively studied. Hence, it is well-established that GPR37 is a substrate for parkin, an E3 ubiquitin ligase involved in the ubiquitination and proteasome-mediated degradation/clearance of misfolded proteins^5^. Indeed, both the loss of function of parkin and its toxic accumulation have been found in some states of Parkinson’s disease (PD)^6–8^. Of note, it was recently described that GPR37 suffers constitutive metalloproteinase-mediated proteolysis, which results on the release of an N-terminal ectodomain^9^. Accordingly, this ecto-domain could be responsible for its toxicity upon overexpression. Alternatively, a role for GPR37 on neuroprotection has been also suggested, since it has been associated with the action of some peptides^10^. Nevertheless, the toxic effects of GPR37 accumulation seem clear in PD. Thus, GPR37 up-regulation, or the presence of GPR37 in Lewy bodies, has been found in brains from PD patients^11–13^. Furthermore, it was shown that the absence of GPR37 in a mouse model of PD (i.e. MPTP) protected against dopaminergic cell death^14^.

The role of GPR37 in PD has been investigated focusing on the interaction of this orphan receptor with different neurotransmitter systems. In such way, it has been shown that GPR37 deletion increases pre-synaptic dopamine transporter (DAT) cell surface expression in the striatum^15^. Similarly, it has been described that the adenosinergic-dependent control of anxiety behavior is modified in GPR37 deficient mice (GPR37−/−)^2^, and that upon deletion of GPR37, adenosine A_2A_ receptors (A_2A_R) antagonists cannot revert pilocarpine-induced tremor, which is a model for parkinsonism^16^. On the other hand, some data point to the existence of direct receptor-receptor interactions involving GPR37. Thus, it was firstly proposed that GPR37 may interact with dopamine D_2_ receptors (D_2_R)^17^. Indeed, it has been recently hypothesized that GPR37 and A_2A_R might form receptor-receptor complexes or heteromers in the striatum^18^, a fact that has not been still wholly demonstrated. Interestingly, the existence of a direct receptor-receptor interaction between A_2A_R and dopamine D_2_ receptor (D_2_R) in the striatum was recently demonstrated and proposed as a pharmacological target for PD management^19^. Hence, it could be postulated that the relevance of GPR37 in PD could be related to its interaction with striatal A_2A_R and/or D_2_R. Supporting this idea, we recently reported that GPR37−/− mice showed lower haloperidol-induced catalepsy^18^, thus suggesting an altered functioning of postsynaptic striatal D_2_R^20^ in GPR37−/− mice. In addition, the well-known effects of A_2A_R antagonists blocking haloperidol-induced catalepsy were higher in the absence of GPR37, thus suggesting a GPR37-dependent A_2A_R modulation of dopaminergic transmission. Overall, GPR37 may play a key role in the A_2A_R-D_2_R interplay and consequently it could be considered as a novel target for PD management.

Here, we aimed to validate the existence of GPR37-A_2A_R oligomers in the striatum and to assess the functional impact of this receptor-receptor interaction *in vivo*. Accordingly, we examined the effects of GPR37 presence/absence on A_2A_R localization (cell surface targeting) and function (cAMP accumulation and behavioral activities, namely catalepsy and locomotor activity).

## Results

### Striatal GPR37 and A_2A_R are enriched at the postsynaptic compartment

**Figure 1:**
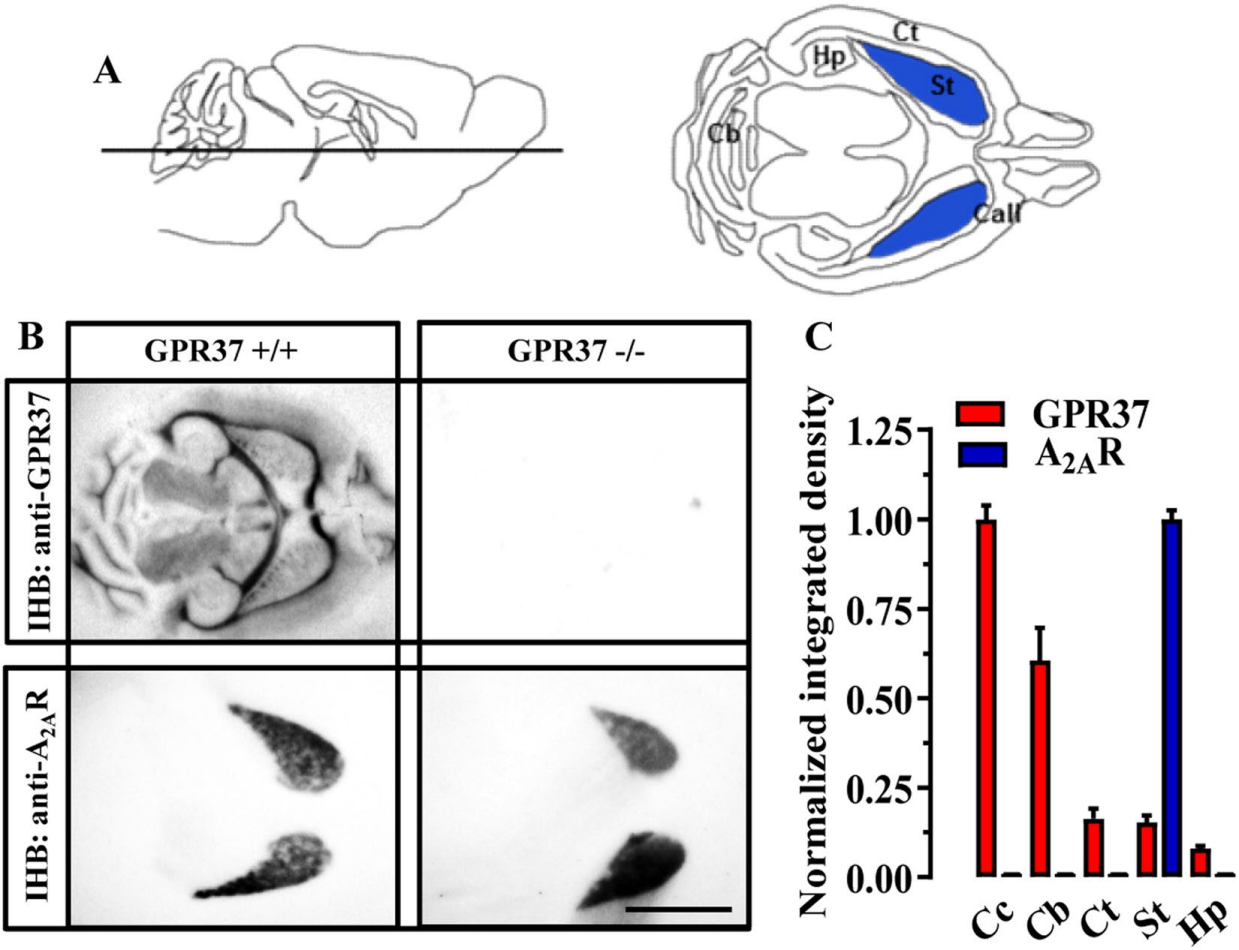
Co-distribution of GPR37 and A_2A_R in the mouse brain. (**A**) Cartoon showing the location of the horizontal brain section for the histoblot (left panel). The expected brain regions within the horizontal section (right panel) are indicated: Cb, cerebellum; Hp, hippocampus; Ct, cortex; St, striatum (blue); Call, corpus callosum. (**B**) Histoblots from horizontal sections (see panel A) of GPR37+/+ and GPR37−/− mouse brain. GPR37 and A_2A_R were detected using a rabbit anti-GPR37-N antibody (3 μg/ml) and a goat anti-A_2A_R antibody (3 μg/ml) (see Materials and Methods). (**C**) The histoblots were scanned and densitometric measurements from six independent experiments were averaged to compare the GPR37 and A_2A_R protein densities. Since expression was maximal in *corpus callosum*, normalization was performed assigning it the 100%. Results are expressed as mean ± SEM. Scale bar: 0.4 cm.

By using a systematic interactome mapping of protein-protein interactions for the A_2A_R, we recently identified and validated GPR37 receptor as a putative interacting partner of A_2A_R^18^. Here, we first aimed to wholly demonstrate and characterize the existence of this direct receptor-receptor interaction (i.e. GPR37-A_2A_R oligomerization) in native tissue. To this end, we first determined the regional and overlapping localization of these two receptors in the mouse brain by using conventional immunohistoblotting. GPR37 was widely distributed throughout the brain with a high degree of expression in myelinated tracts including *corpus callosum* and cerebellar white matter tracts (Fig. 1A), as previously described by means of *in situ* hybridization^4^. In addition, a moderate to weak labeling was consistently detected in cortex, striatum and hippocampus (Fig. 1A and B). Interestingly, no immunostaining was observed when brain sections from GPR37−/− were used (Fig. 1A), thus demonstrating the specificity of the antibody used (i.e. rabbit anti-GPR37-N). Finally, under the same experimental conditions we found that A_2A_R expression was mostly concentrated in the striatum (Fig. 1), as expected^21, 22^. Overall, these first experiments pinpointed the striatum as a brain region to assess GPR37-A_2A_R interaction.

**Figure 2:**
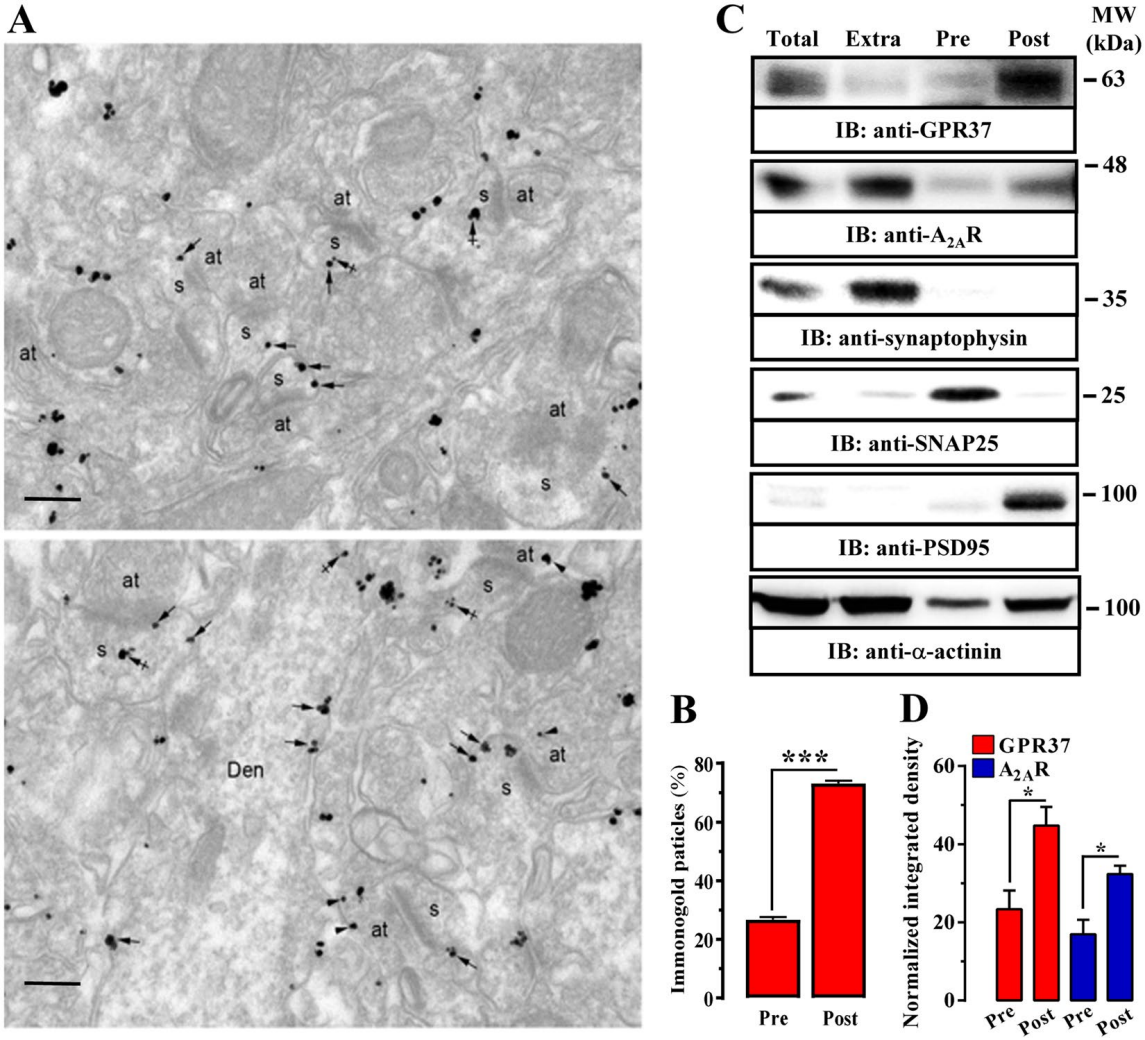
Subsynaptic distribution of GPR37 in the mouse striatum. (**A**) Electron micrographs showing immunoparticles for GPR37 in the striatum of GPR37+/+ mice using the pre-embedding immunogold technique. GPR37 immunoparticles were abundant on the extrasynaptic plasma membrane (arrows) of dendritic spines (s) of striatal neurons contacted by axon terminals (at). Few immunoparticles were observed at intracellular sites (crossed arrows) in dendritic spines (s) (upper panel). Immunoparticles for GPR37 were also localized to the extrasynaptic plasma membrane (arrowheads) of axon terminals (at) establishing asymmetrical synapses with spines (s). In dendritic shafts (Den), immunoparticles for GPR37 were mainly found at the plasma membrane (arrows) (lower panel). Scale bars: 200 nm. (**B**) Bar graphs showing the percentage of GPR37 immunoparticles at post- and presynaptic compartments. The data are expressed as mean ± SEM of three independent experiments (***P < 0.001; Student’s *t*-test). (**C**) Representative immunoblots showing GPR37 and A_2A_R immunoreactivity in striatal synaptic fractions. Striatal synaptosomes (Total) were subcellularly fractionated (see Materials and Methods section) into extrasynaptic (Extra), presynaptic active zone (Pre) and postsynaptic density (Post) fractions, which were analyzed by SDS-PAGE (20 μg of protein/lane) and immunoblotted using rabbit anti-GPR37-N, goat anti-A_2A_R, rabbit anti-synaptophysin, mouse anti-PSD-95, mouse anti-SNAP-25 and rabbit anti-α-actinin antibodies. The primary antibodies were detected using a horseradish peroxidase (HRP)-conjugated goat anti-rabbit IgG, HRP-conjugated goat anti-mouse IgG, HRP-conjugated rabbit anti-goat IgG and chemiluminescence detection (see Materials and Methods). (**D**) Relative quantification of GPR37 enrichment in striatal presynaptic and postsynaptic fractions. The intensities of the immunoreactive bands on the immunoblotted membranes corresponding to extrasynaptic, presynaptic (Pre) and postsynaptic (Post) fractions were measured by densitometric scanning. Values were first normalized to the loading control (i.e. α-actinin) and then to the amount of GPR37 or A_2A_R in the total fraction. Data are expressed as mean ± SEM of three independent experiments (*P < 0.05, Student’s *t*-test).

Next, we aimed to ascertain the subsynaptic striatal locus where a putative interaction between these two receptors might occur. We first evaluated the subcellular localization of GPR37 within the striatum by means of immunogold electron microscopy. GPR37 immunoparticles were mostly found at the extrasynaptic plasma membrane of dendritic spines of striatal neurons (Fig. 2A, arrows), with few immunoparticles present at intracellular sites (Fig. 2A, crossed arrows). Similarly, in axon terminals establishing asymmetrical synapses with striatal spines GPR37 immunoparticles were also localized at the extrasynaptic plasma membrane (Fig. 2A, lower panel, arrowheads). Finally, in dendritic shafts, immunoparticles for GPR37 were mainly found at the plasma membrane (Fig. 2A, lower panel arrows). Interestingly, quantitative analysis showed that 26 ± 0.9% of immunoparticles were located presynaptically while 73 ± 0.9% showed a postsynaptic distribution (Fig. 2B), thus matching with A_2A_R distribution within the striatum^21^. Subsequently, we performed a subsynaptic fractionation of striatal nerve terminals, which allowed identifying the localization of GPR37 and A_2A_R^23, 24^ in pre-, post- and extrasynaptic enriched fractions (Fig. 2C). Interestingly, immunoblot analysis of the different striatal subsynaptic fractions revealed a significant (*P* < 0.05) enrichment at the postsynaptic over the presynaptic fraction of both GPR37 and A_2A_R (Fig. 2C and D), thus showing their superimposable subsynaptic distribution within the striatum. On the other hand, we ascertained the well-characterized high A_2A_R extrasynaptic expression, which has been reported to be mostly related to the actions of adenosine acting as an energy-dependent neuromodulator in the SNC, also finely sensoring neuronal activity^25^.

### Co-clustering of GPR37 and A_2A_R in striatum

**Figure 3:**
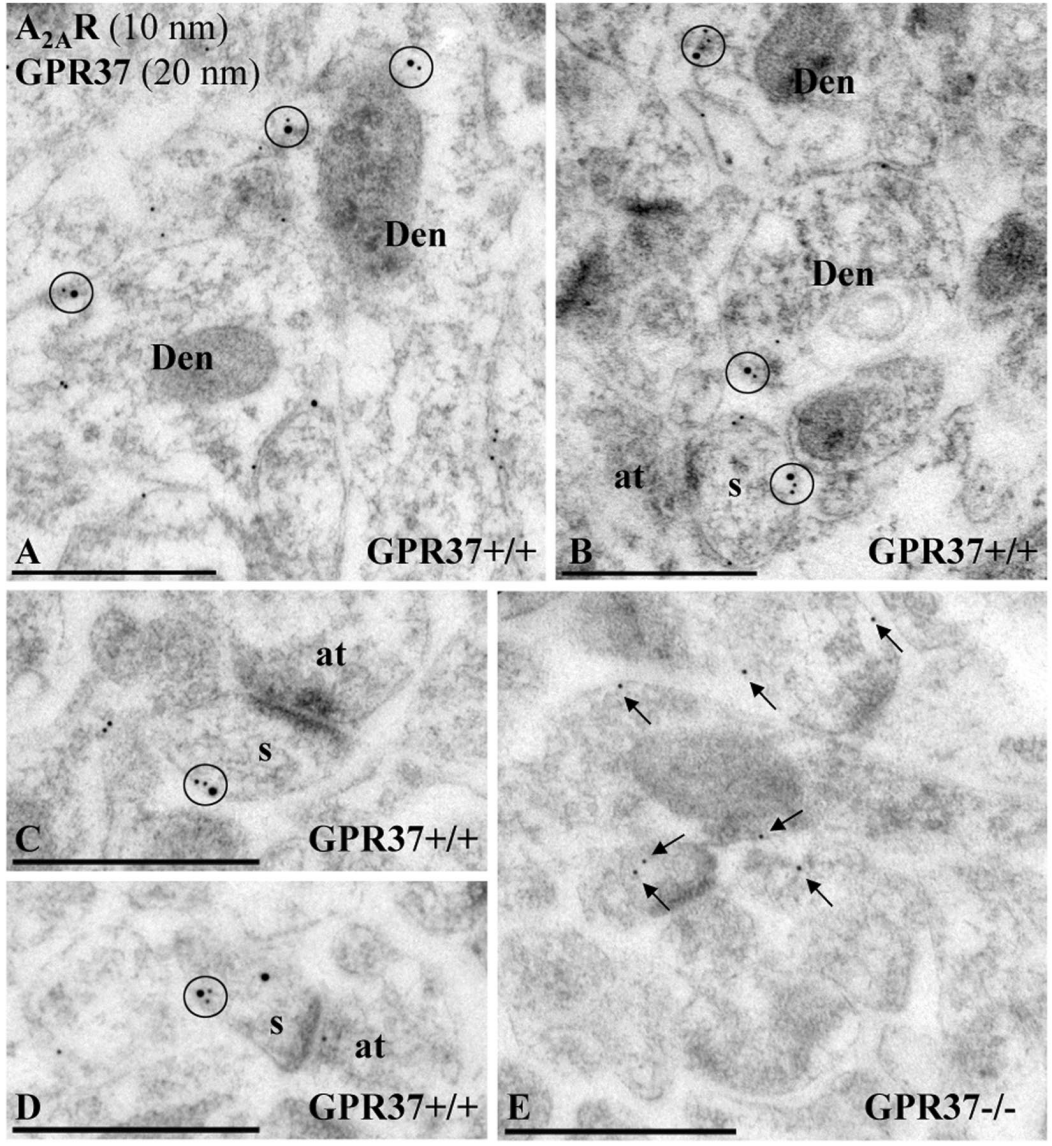
Post-embedding electron microscopy showing GPR37 and A_2A_R co-localization in the mouse striatum. Electron micrographs from GPR37+/+ and GPR37−/− showing immunoreactivity for GPR37 and A_2A_R in the striatum revealed using a double-labelling post-embedding immunogold technique. (**A–D**) In GPR37+/+ mice, immunoparticles for A_2A_R (10 nm size) and GPR37 (20 nm size) were closely co-distributed (circles) along the extrasynaptic plasma membrane of dendritic shafts (Den) and dendritic spine (s) establishing excitatory synaptic contact with axon terminals (b). (**E**) In GPR37−/− mice, immunoparticles for A_2A_R (10 nm size) are present in the tissue but not immunoparticles for GPR37, demonstrating the full specificity of the anti-GPR37-N antibody. Scale bars: A–E, 500 nm.

While biochemical approaches are useful to establish the subsynaptic distribution of neuronal proteins they do not provide spatial information. Thus, we aimed to determine the proximity between GPR37 and A_2A_R within striatal neurons by means of double-labelling immunogold electron microscopy and proximity ligation *in situ* assay (P-LISA). First, it was observed that GPR37 and A_2A_R closely co-distributed along the extrasynaptic plasma membrane of dendritic shafts and dendritic spines establishing excitatory synaptic contact with axon terminals (Fig. 3A–D). This result agrees with the previously findings reported in the subsynaptic fractionation experiments. Importantly, in the striatum of GPR37−/− mice, no GPR37 detection was observed (Fig. 3E), again demonstrating the specificity of the anti-GPR37-N antibody used. Overall, these results revealed a close and selective anatomical proximity of GPR37 and A_2A_R within striatal spines.

**Figure 4:**
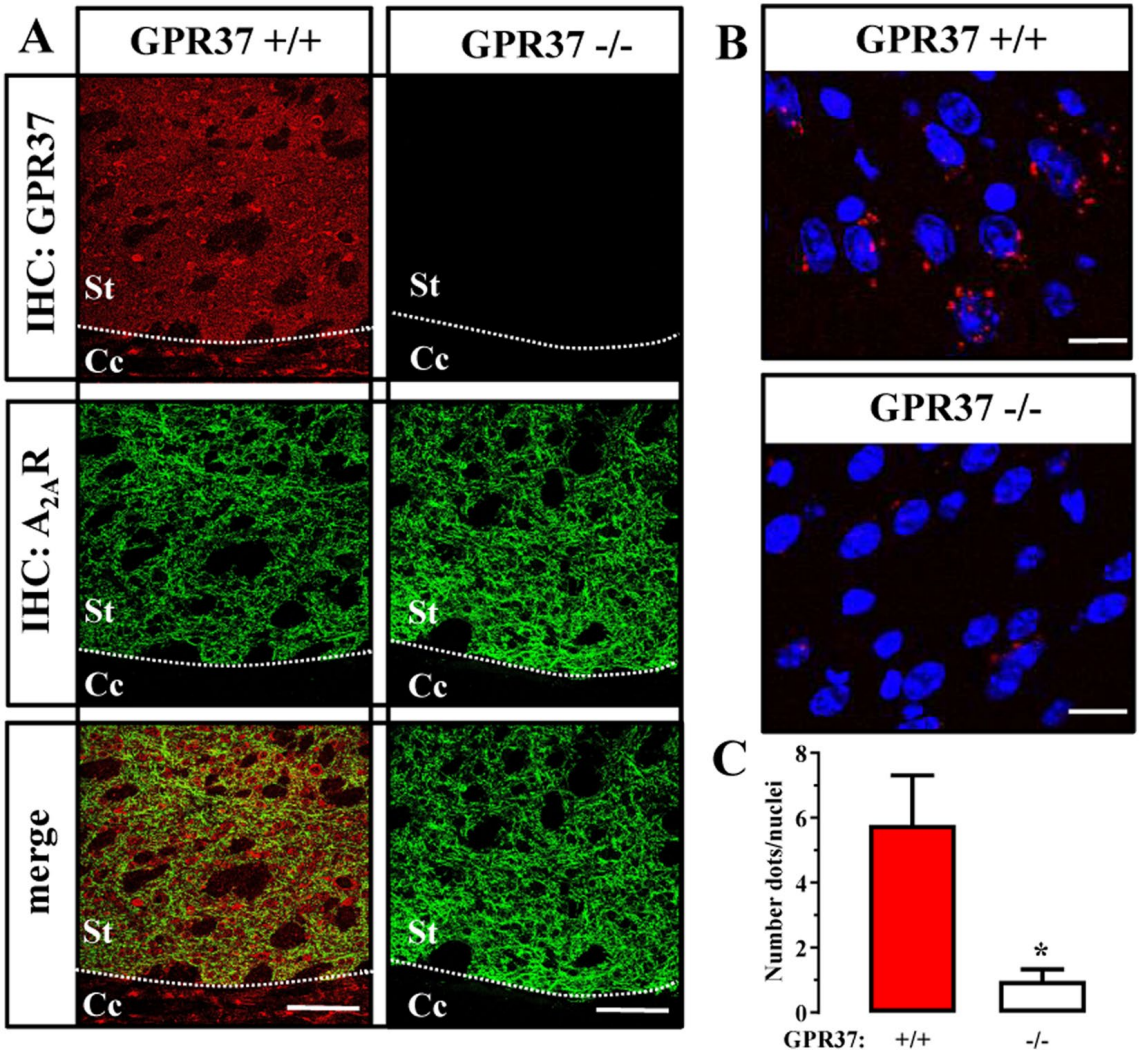
GPR37 and A_2A_R interact in the striatum. (**A**) Representative images of GPR37 and A_2A_R immunoreactivities in the dorsal striatum of GPR37+/+ and GPR37−/− mice. Superimposition of images revealed receptor co-distribution in yellow (merge). Scale bar: 350 μm. Cc, corpus callossum; St; striatum. (**B**) Photomicrographs of dual recognition of GPR37 and A_2A_R with P-LISA in striatal sections from GPR37+/+ and GPR37−/− mice. Scale bar: 10 μm. (**C**) Quantification of P-LISA signals for GPR37 and A_2A_R proximity in GPR37+/+ and GPR37−/−. Values in the graph correspond to the mean ± SEM (dots/nuclei) of at least five animals for each condition. Asterisk indicates statistically significant differences (*p* < 0.05; Student’s *t*-test) when comparing GPR37−/− with GPR37+/+.

We next aimed to validate the existence of GPR37/A_2A_R heteromers in the striatum by means of P-LISA, a well described technique that provides enough sensitivity to evaluate receptor’s close proximity within a named GPCR oligomer in native conditions^26^. We first ascertained the immunohistochemistry detection of GPR37 and A_2A_R in the striatum. As expected, both receptors showed a high degree of co-distribution throughout the striatal neuropil (Fig. 4A). Interestingly, no immunostaining was observed when brain slices from GPR37−/− were used (Fig. 1A, upper right panel), thus demonstrating the specificity of the antibody used (i.e. rabbit anti-GPR37-C). Noteworthy, here we used an antibody directed against the C-terminus of GPR37. This is a mandatory requirement for the PLA assay, in which both antibodies must recognize the extracellular or intracellular epitopes of the selected putative interacting proteins. Subsequently, we implemented the P-LISA approach, using an adequate combination of antibodies to test the presence of GPR37/A_2A_R heteromers in the mouse striatum. Notably, red dots reflecting a positive P-LISA signal were observed in the striatum of GPR37+/+ mice (Fig. 4B, upper panel), thus allowing the visualization of the GPR37/A_2A_R receptor-receptor interaction. Importantly, in striatal slices from GPR37−/− mice the P-LISA signal was negligible (Fig. 4B, lower panel), thus reinforcing the specificity of our P-LISA assay. Indeed, when the P-LISA signal was quantified, 6.5 ± 1.6 dots/nuclei were observed in GPR37+/+ mice, while GPR37−/− mice only displayed 0.9 ± 0.3 dots/nuclei under the same experimental conditions. Thus, a marked and significant (*P* < 0.05) reduction in the P-LISA signal was observed in GPR37−/− striatal slices, which strongly supported the existence of GPR37/A_2A_R heteromers in the mouse striatum.

### GPR37 deletion promotes striatal A_2A_R cell surface expression and function

**Figure 5:**
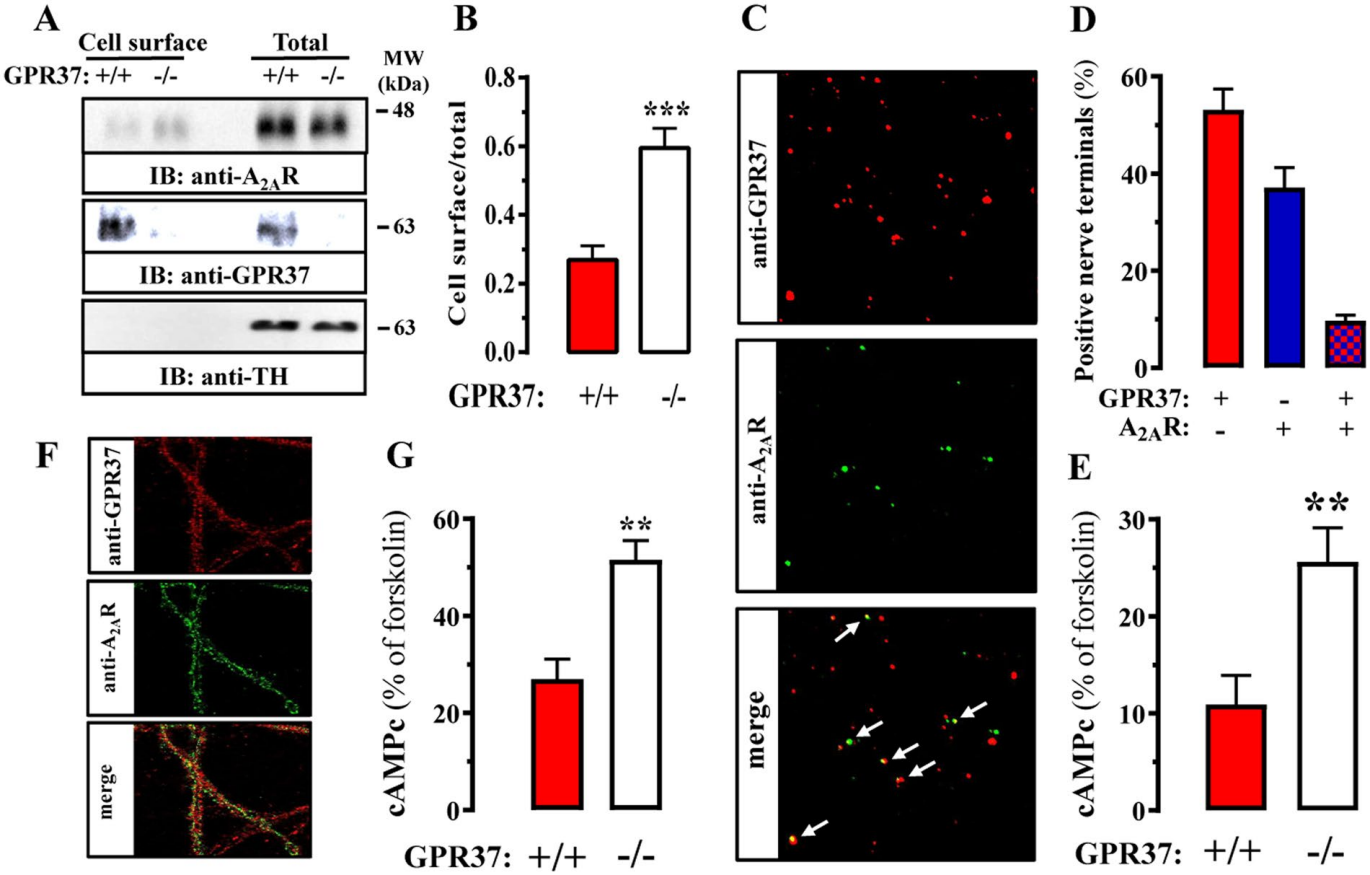
GPR37 deletion bolsters striatal A_2A_R cell surface expression and function. (**A**) Coronal brain slices (300 µm) from GPR37+/+ and GPR37−/− mice were prepared and biotinylated as described in Material and Methods section. Total and cell surface extracts were analyzed by SDS-PAGE and immunoblotting as described in Fig. 2. (**B**) Quantification of A_2A_R cell surface density in GPR37+/+ and GPR37−/− striatum. Cell surface density was normalized using the total density of A_2A_R and expressed as mean ± SEM from six independent experiments. The asterisk indicates statistically significant difference from the control condition (*P* < *0.05;* paired Student’s *t* test). (**C**) Co-distribution of GPR37 and A_2A_R in striatal synaptosomes. Immunofluorescence detection of GPR37 (red) and A_2A_R (green) in striatal total synaptosomes was performed as described in Materials and Methods. Superimposition of images (merge) reveals co-localization in yellow (arrows). (**D**) Quantification of synaptosomes expressing GPR37 and/or A_2A_R. The data are expressed as the percentage (mean ± SEM) of total number of synaptosomes that are endowed with GPR37 and/or A_2A_R, quantified in 3–4 different synaptosomal preparations from different mice, in which four different fields acquired from two different coverslips were analysed in each preparation. (**E**) A_2A_R-mediated cAMP accumulation in synaptosomes. Total striatal synaptosomes from GPR37+/+ and GPR37−/− mice were stimulated with 500 nM CGS21680 for 30 min at 37 °C and the cAMP accumulation was measured as described in Materials and Methods. The data are expressed as percentage (mean ± SEM) of forskolin-induced cAMP accumulation from eight independent experiments, which was similar (P > 0.05) in both genotypes. The asterisks indicate statistically significant difference from the control condition (*P* < *0.01*; Student’s *t* test). (**F**) Co-distribution of GPR37 and A_2A_R in striatal neurons. Immunofluorescence detection of GPR37 (red) and A_2A_R (green) in primary cultured striatal neurons (DIV21) was performed as described in Materials and Methods. Superimposition of images (merge) reveals co-localization in yellow. Scale bar: 100 μm. (**G**) A_2A_R-mediated cAMP accumulation in striatal neurons. Striatal primary neurons from GPR37+/+ and GPR37−/− were stimulated with 500 nM CGS21680 for 30 min at 37 °C and the cAMP accumulation was measured as described in Materials and Methods. The data are expressed as percentage (mean ± SEM) of forskolin-induced cAMP accumulation from five independent experiments, which was similar (P > 0.05) in both genotypes. The asterisks indicate statistically significant difference from the control condition (*P* < *0.01*; Student’s *t* test).

It has been shown that GPR37 cell surface expression in cultured cells can be regulated by co-expressing particular GPCRs, including the A_2A_R^17^. Indeed, we recently demonstrated that GPR37 and A_2A_R form heteromers *in vitro* with an intertwined cell surface expression when co-expressed^18^. However, the function of the GPR37-A_2A_R interaction in native tissue is still enigmatic. Therefore, we aimed to ascertain the impact of GPR37 expression on A_2A_R trafficking and function in native tissues. To this end, we first assessed A_2A_R cell surface expression by means of biotynilation of striatal slices, thus enabling the selective quantification of surface expressed receptors in GPR37+/+ and GPR37−/− mice. Interestingly, while total A_2A_R density was unaltered, the A_2A_R cell surface density was increased in the striatum of GPR37−/− mice (Fig. 5A and B). Of note, the absence of tyrosine hydroxylase immunoreactivity in the biotinylated surface fractions indicated that the integrity of slices was maintained and no major cell damage occurred during striatal slice preparation. Thus, our results demonstrated that GPR37 deletion potentiates A_2A_R cell surface expression.

Next, we examined whether the increased A_2A_R cell surface expression in GPR37−/− striatum translated into an increased A_2A_R function. To this end, we evaluated A_2A_R-dependent cAMP accumulation in striatal nerve terminals from GPR37+/+ and GPR37−/− mice. First, we undertook a complementary double immunocytochemistry study in individual striatal synaptosomes to identify synapses endowed with both GPR37 and A_2A_R (Fig. 5C). As illustrated in Fig. 5D, we observed that around 9 ± 1.1% of total population of striatal synaptosomes (immunopositive for both SNAP25 and PSD95), presented immunoreactivity for both GPR37 and A_2A_R (Fig. 5D). Subsequently, we compared the ability of an A_2A_R agonist to trigger cAMP accumulation in striatal synaptosomes from GPR37+/+ and GPR37−/− mice. A significant 2.5-fold increase in A_2A_R-mediated cAMP accumulation was observed in synaptosomes from GPR37−/− mice (Fig. 5E). Finally, similar A_2A_R-based functional assays were performed in striatal primary neurons from GPR37+/+ and GPR37−/− mice (Fig. 5F). Again, a significant increase in A_2A_R-mediated cAMP accumulation was observed in primary cultures from GPR37−/− striatum (Fig. 5G). Overall, these results demonstrated that GPR37 deletion bolstered striatal A_2A_R cell surface expression as well as A_2A_R function.

### GPR37 deletion enhances A_2A_R function *in vivo*

**Figure 6:**
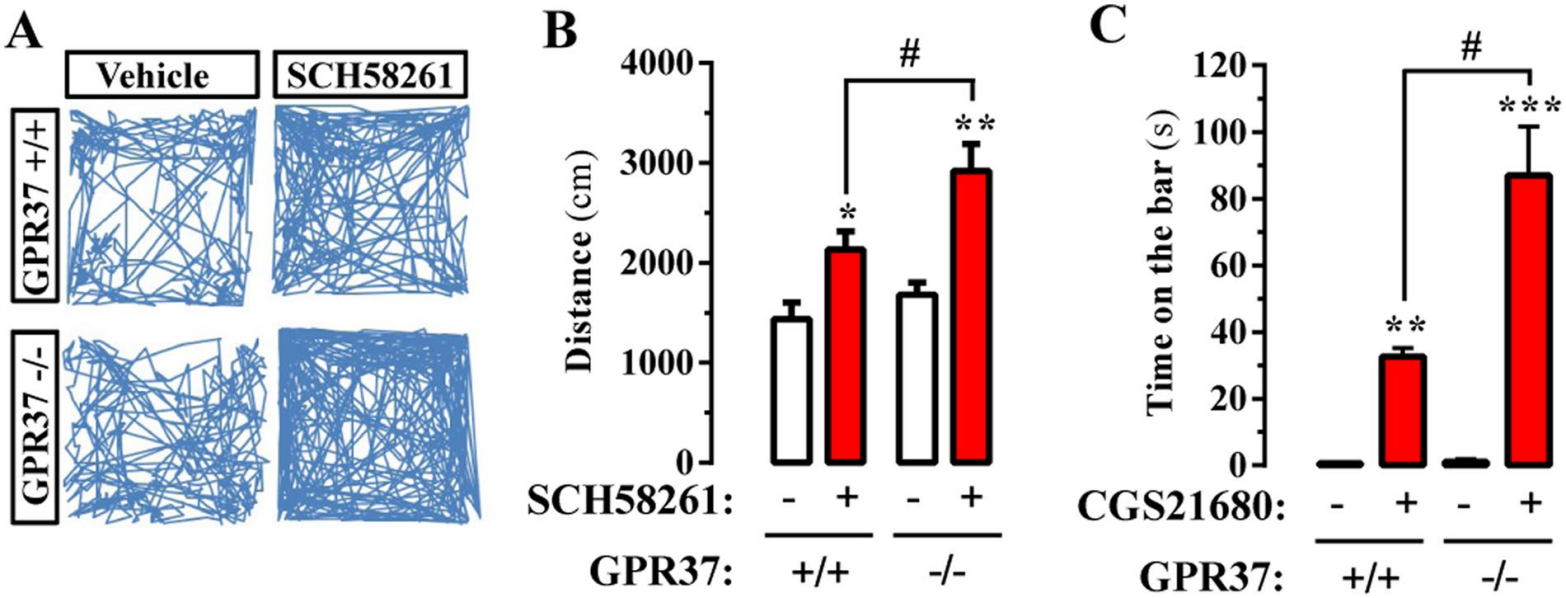
GPR37 deletion promotes A_2A_R-mediated behavior. (**A**) A_2A_R antagonist-induced locomotor hyperactivity in GPR37+/+ and GPR37−/− mice. Representative 10 min trajectories in an open field arena of GPR37+/+ and GPR37−/− mice administered intraperitoneally with vehicle or SCH58261 (3.75 mg/kg). (**B**) Quantification of the horizontal locomotor activity shown in (**A**). The distance travelled is expressed as mean ± SEM (n = 10 animals) *P < 0.05, **P < 0.01 SCH58261 treatment effect (one-way ANOVA, followed by Tukey post-hoc test), ^#^ *P* < 0.05 phenotype effect (two-way ANOVA, Bonferroni post-hoc test). (**C**) A_2A_R agonist-induced catalepsy in GPR37+/+ and GPR37−/− mice. The cataleptic response induced by intracerebroventricular administration of CGS21680 (10 µl of a 1 µg/µl solution) in GPR37+/+ and GPR37−/− mice was measured as the duration of an abnormal upright posture in which the forepaws of the mouse were placed on a horizontal wooden bar. The time spent with both front paws resting on the bar is expressed as mean ± SEM (n = 10 animals); a cut off time was set at 200 s. **P < 0.01, ***P < 0.001 CGS21680 treatment effect (one-way ANOVA, followed by Tukey post-hoc test); ^#^ *P* < 0.05 phenotype effect (Two-way ANOVA, Bonferroni post-hoc test).

Once we confirmed an enhanced striatal A_2A_R activity in GPR37−/− mice, we aimed to determine its impact in animal behavior. It is well established that striatal A_2A_R modulates the central processes involved in locomotor activity and psychomotor behaviors due to its interplay with the dopaminergic system^27^. Our data predicted an enhanced modulation of locomotor activity by striatal A_2A_R in GPR37−/− mice. Since it is well-known that blocking of A_2A_R increases spontaneous locomotor activity in mice^28^, we compared the hyper-motility induced by an A_2A_R antagonist in GPR37+/+ and GPR37−/− mice. To this end, we challenged GPR37+/+ and GPR37−/− mice with SCH58261 and the spontaneous locomotor activity in an open field arena was monitored (Fig. 6A). Our results showed that A_2A_R antagonist-mediated locomotor activity potentiation was significantly (*P* < 0.05) higher in GPR37−/− mice (Fig. 6B), thus suggesting an increased A_2A_R basal activity in the absence of GPR37.

Conversely to hyperlocomotion induced by A_2A_R antagonists, activation of striatal A_2A_R with selective agonists can suppress motor activity, thus producing effects that resemble those yielded by D_2_R antagonists (i.e. haloperidol) or dopamine depletion (i.e. reserpine)^29^. For instance, administration of the A_2A_R agonist CGS 21680 blocked acquisition and expression of wheel running behavior^30^, depressed locomotor activity^31^ and induced catalepsy^32^. We now assessed whether A_2A_R agonist-induced catalepsy was increased in GPR37−/− mice. Our results showed that upon CGS21680 administration (i.c.v.) the catalepsy scores of GPR37−/− mice were significantly higher (*P* < 0.001) than these observed in GPR37+/+ mice (Fig. 6C), thus demonstrating an increased activity of striatal A_2A_R in GPR37−/− mice. Overall, our results suggested that GPR37 may modulate A_2A_R control of locomotor activity and psychomotor behavior through a putative GPR37/A_2A_R heteromer. Needless to say, as commented previously, GPCR display the ability to oligomerize, but receptors may also exist in their monomeric form. Accordingly, the observed effects of A_2A_R ligands on locomotor activity and psychomotor activity would be interpreted within this more complex scenario, in which different populations of receptors exist, forming or not heteromers.

## Discussion

GPR37 is an orphan GPCR highly expressed throughout the brain. Although it has been related to the dopaminergic system^15^ and brain myelination^4^, the physiological role of this receptor has not been fully elucidated. Recently, by using a modified membrane yeast two-hybrid (MYTH) approach, GPR37 was identified as a putative A_2A_R interacting partner^18^, prompting the exploration of the biological relevance of this interaction in the native brain. Our experimental data strongly supports the existence of GPR37/A_2A_R heteromers in the mouse striatum. Thus, the former GPR37-A_2A_R interaction picked up *in vitro* by MYTH^18^ was further validated in native tissue by double-labelling immunogold electron microscopy and P-LISA, which permitted to definitely establish the existence of a close proximity between these two receptors in the mouse striatum. These findings prompted us to assess the impact of this oligomer in animal behavior. Accordingly, our main objective consisted of unravelling the *in vivo* consequences of GPR37/A_2A_R heteromer formation, the fingerprint for any named GPCR oligomer^33^. Thus, while we can’t rule out existence of a heteromer-independent functional interplay, the following findings shed light regarding the *in vivo* function of the newly described striatal GPR37/A_2A_R heteromer: i) GPR37 and A_2A_R displayed a high and selective anatomical proximity within striatal spines; ii) GPR37 deletion potentiated A_2A_R cell surface targeting in striatal slices; iii) A_2A_R-mediated signaling was enhanced in striatal synaptosomes and primary cultures from GPR37−/−; iv) GPR37 deletion potentiated A_2A_R antagonist-mediated locomotor activity and A_2A_R agonist-induced catalepsy. Based on these data, we concluded that GPR37 is a negative modulator of A_2A_R cell surface expression and function *in vivo*, and whether the formation of the GPR37/A_2A_R heteromer is required for these effects should be further explored in the near future.

The striatum, the main input structure of the basal ganglia^34^, is responsible for both movement and learning/reward behavior. Importantly, dopaminergic projections from the substantia nigra pars compacta (SNc) are essential for motor control and their degeneration in PD results in severe motor problems^35^. Indeed, conventional therapies for PD are primarily devoted to replacing and support dopaminergic neurotransmission. In such way, early PD motor symptoms respond well to dopamine-based therapies including levodopa and D_2_R agonists^36^. However, in the long term, these agents may lead to the development of serious motor complications that limit their efficacy^37^. These therapeutic obstacles have prompted the search for alternative therapies based on novel nondopaminergic drug^38^. A new therapeutic target is the adenosinergic system. In fact, A_2A_R antagonists have not only been postulated but they have also been licensed as antiparkinsonian drugs^39^. Importantly, it is believed that the A_2A_R/D_2_R heterodimer may underlie the observed antiparkinsonian effects of A_2A_R antagonists^40^. In addition, the striatal A_2A_R/D_2_R heterodimer has been shown to be downregulated in experimental parkinsonism^19^, a fact that parallels a concomitant increase in A_2A_R constitutive activity^28^.

The ability of striatal GPR37 to oligomerize with A_2A_R, and possibly with D_2_R, might constitute a way for fine-tuning multiple receptor-signaling pathways and harmonizing dopaminergic neurotransmission. Certainly, manipulating the GPR37/A_2A_R heteromer stoichiometry could impact on D_2_R functioning through putative postsynaptic GPR37/D_2_R/A_2A_R-containing complexes present in GABAergic striatopallidal neurons. Hence, it could be hypothesized that an increased GPR37/A_2A_R oligomerization would repress A_2A_R activity and, therefore, indirectly rise D_2_R function, mimicking the effects of a direct activation of the receptor. This last hypothesis still needs to be experimentally probed, but it seems clear that the GPR37/A_2A_R heteromer, which here we demonstrate is formed in native conditions, may constitute a novel and very attractive target for the design of new pharmacological strategies to manage pathologies affecting dopaminergic neurotransmission such as PD.

## Methods

### Animals

C57BL/6J wild type (GPR37+/+) and GPR37 deficient (GPR37−/−) male mice (Strain Name: B6.129P2-*GPR37* ^*tm1Dgen*^/J; The Jackson Laboratory, Bar Harbor, ME, USA) with 8 weeks of age were used^2^. The University of Barcelona Committee on Animal Use and Care approved the protocol. Animals were housed and tested in compliance with the guidelines described in the Guide for the Care and Use of Laboratory Animals^41^ and following the European Union directives (2010/63/EU). All efforts were made to minimize animal suffering and the number of animals used. All animals were housed in groups of five in standard cages with *ad-libitum* access to food and water and maintained under 12 h dark/light cycle (starting at 7:30 AM), 22 °C temperature, and 66% humidity (standard conditions).

### Immunohistoblotting

The regional distribution of GPR37 and A_2A_R was analyzed in the C57BL6 mouse brain using an *in situ* blotting technique^42, 43^. Briefly, horizontal cryostat sections (25 µm) were placed on nitrocellulose membranes 0.45 µm (Whatman) moistened with 48 mM Tris-base, 39 mM glycine, 2% (w/v) SDS and 20% (v/v) methanol for 15 min at room temperature (~20 °C). After blocking in 5% (w/v) non-fat dry milk in phosphate-buffered saline, the nitrocellulose membranes were treated with Deoxyribonuclease I from bovine pancreas (DNase I, 5 U/mL, Sigma-Aldrich, St. Louis, MO, USA), washed and incubated in 2% (w/v) SDS and 100 mM β-mercaptoethanol in 100 mM Tris–HCl (pH 7.0) for 60 min at 45 °C to remove adhering tissue residues. After extensive washing, the blots were incubated overnight at 4 °C with rabbit anti-GPR37-N polyclonal antibody (3 µg/ml)^2^ or goat anti-A_2A_R polyclonal antibody (AB_2571655; 3 µg/ml; Frontier Institute Co. Ltd, Shinko-nishi, Ishikari, Hokkaido, Japan) in blocking solution. The bound primary antibodies were detected with alkaline phosphatase-conjugated secondary antibodies. All nitrocellulose membranes were processed in parallel, using the same incubation times and antibody/reagent concentrations. Digital images were acquired with a Stereo Lumar.v12.

### Fixed brain tissue preparation

Mice were anesthetized and perfused intracardially with 100 ml of ice-cold 4% paraformaldehyde (PFA) in phosphate buffered saline (PBS; 8.07 mM Na_2_HPO_4_, 1.47 mM KH_2_PO_4_, 137 mM NaCl, 0.27 mM KCl, pH 7.2). Brains were post-fixed overnight in the same solution of PFA at 4 °C. Coronal sections (50 μm) were obtained using a vibratome (Leica Lasertechnik GmbH, Heidelberg, Germany). Slices were collected in Walter’s Antifreezing solution (30% glycerol, 30% ethylene glycol in PBS, pH 7.2) and kept at −20 °C until processing.

### Immunoelectron microscopy

#### Pre-embedding immunogold technique

Immunohistochemical reactions for electron microscopy were carried out using the pre-embedding immunogold method described previously^44^. Briefly, free-floating sections were incubated in 10% (v/v) NGS (normal goat serum) diluted in TBS. Sections were then incubated with rabbit anti-GPR37-N (3–5 μg/ml diluted in TBS containing 1% (v/v) NGS), followed by incubation in goat anti-rabbit IgG coupled to 1.4 nm gold (Nanoprobes Inc., Stony Brook, NY, USA). Sections were post-fixed in 1% (v/v) glutaraldehyde and washed in double-distilled water, followed by silver enhancement of the gold particles with an HQ Silver kit (Nanoprobes Inc.). Sections were then treated with osmium tetraoxide (1% in 0.1 m phosphate buffer), block-stained with uranyl acetate, dehydrated in graded series of ethanol and flat-embedded on glass slides in Durcupan (Fluka, Sigma-Aldrich) resin. Regions of interest were cut at 70–90 nm on an ultramicrotome (Reichert Ultracut E, Leica, Austria) and collected on single slot pioloform-coated copper grids. Staining was performed on drops of 1% aqueous uranyl acetate followed by Reynolds’s lead citrate. Ultrastructural analyses were performed in a Jeol-1010 electron microscope. To study the frequency of GPR37, we counted immunoparticles identified in each reference area and present in different subcellular compartments: dendritic spines, dendritic shafts and axon terminals. The data were expressed as a percentage of immunoparticles in each subcellular compartment, both in the plasma membrane and at intracellular sites.

#### Post-embedding immunogold technique

To study the spatial relationship between A_2A_R and GPR37 we used double-labelling post-embedding immunogold techniques, as previously described^45^. Briefly, ultrathin sections 80-nm thick from Lowicryl-embedded blocks of striatum were picked up on coated nickel grids and incubated on drops of a blocking solution consisting of 2% human serum albumin (HSA) in 0.05 M TBS and 0.03% Triton X-100 (TBST). The grids were incubated with a mixture of goat anti-A_2A_R and rabbit anti-GPR37-N (10 μg/ml in TBST with 2% HSA)^2^ at 28 °C overnight. The grids were incubated on drops of rabbit anti-goat IgG or goat anti-rabbit IgG conjugated to 10 nm and 20 nm colloidal gold particles, respectively (BBI Solutions, Cardiff, UK) in 2% HSA and 0.5% polyethylene glycol in TBST. The grids were then washed in TBS and counterstained for electron microscopy with saturated aqueous uranyl acetate followed by lead citrate. Ultrastructural analyses were performed in a Jeol-1010 electron microscope. Randomly selected areas were then photographed from the selected ultrathin sections at a final magnification of 50,000 x.

### Gel electrophoresis and immunoblotting

Sodium dodecyl sulfate–polyacrylamide gel electrophoresis (SDS/PAGE) was performed using 10% polyacrylamide gels. Proteins were transferred to polyvinylidene difluoride membranes using a semi-dry transfer system (Bio-Rad, Hercules, CA, USA) and immunoblotted using rabbit anti-GPR37-N (1 μg/ml)^2^, goat anti-A_2A_R (AB_2571655; 1 μg/ml; Frontier Institute Co. Ltd), rabbit anti-synaptophysin (ab23754; 1 μg/ml; Abcam), mouse anti-PSD-95 (ab13552; 1 μg/ml; Abcam), mouse anti-SNAP-25 (ab66066; 1 μg/ml; Abcam) and rabbit anti-α-actinin (sc-15335; 0.5 μg/ml; Santa Cruz Biotechnology Inc., Dallas, TX, USA) antibodies. The primary antibodies were detected using a horseradish peroxidase (HRP)-conjugated goat anti-rabbit IgG (65–6120; 1/50,000; Pierce Biotechnology), HRP-conjugated goat anti-mouse IgG (31430; 1/20,000; Pierce Biotechnology), HRP-conjugated rabbit anti-goat IgG (61–1620; 1/20,000; Pierce Biotechnology). The immunoreactive bands were developed using a chemiluminescent detection kit (Thermo Fisher Scientific, Waltham, MA, USA) and detected with an Amersham Imager 600 (GE Healthcare Europe GmbH, Barcelona, Spain)^46^.

### Striatal slice biotinylation

Biotinylation is a useful tool for the covalent labelling and separation of cell-surface-expressed proteins in brain slices^47^. In brief, mouse brain was rapidly removed and immediately chilled in sucrose-supplemented artificial CSF (SACSF; 2.5 mM KCl, 1.2 mM NaH_2_PO_4_, 1.2 mM MgCl_2_, 2.4 mM CaCl_2_, 26 mM NaHCO_3_, 11 mM glucose, and 250 mM sucrose) saturated with 95%O_2_/5%CO_2_. Brains were mounted on a Leica vibratome 1200S sectioning system (Leica) and 300 µm coronal sections were made. Sections containing the striatum were recovered in ACSF (125 mM NaCl, 2.5 mM KCl, 1.2 mM NaH_2_PO_4_, 1.2 mM MgCl_2_, 2.4 mM CaCl_2_, 26 mM NaHCO_3_, and 11 mM glucose) at 31 °C, during 90 min with 95% O_2_/5% CO_2_, then were immediately used. Surface proteins were covalently labeled with 0.5 mg/ml sulfo-NHS-SS-biotin in ice-cold ACSF for 45 min at 4 °C, with continuous 95%O_2_/5%CO_2_ bubbling. Following biotinylation, slices were washed 3 times in ice-cold ACSF and three times in ice-cold ACSF supplemented with 100 mM glycine. Residual reactive biotin was quenched by incubating twice in ice-cold ACSF supplemented with glycine during 25 min at 4 °C with continuous 95%O_2_/5%CO_2_ bubbling. Subsequently, slices were washed four times with ice-cold ACSF, triturated with iced-cold radio-immuno assay (RIPA) buffer (150 mM NaCl, 1% NP-40, 50 mM Tris, 0.5% sodium deoxycholate, and 0.1% SDS, pH 8.0) using a Pasteur pipette and rotated in an end over end rotator during 30 min at 4 °C. Cellular debris were cleared by centrifugation at 18,000 × *g* for 15 min at 4 °C and the protein concentration was determined using the bicinchoninic acid (BCA) protein assay (Pierce Biotechnology, Rockford, IL, USA). Next, 25 µl streptavidin agarose were added to 50 µg striatal lysate in 1 ml RIPA buffer and incubated overnight with constant rotation at 4 °C. Finally, streptavidin beads were washed five times with RIPA buffer and resuspended SDS-PAGE sample buffer for immunoblot assay. A rabbit anti-tyrosine hydroxylase antibody (AB152; Merck Millipore, Darmstadt, Germany) was used to ensure proper cell-surface-protein isolation.

### Synaptosomal preparation and subsynaptic fractionation

For synaptosomal preparation the striatum from 6 mice were dissected and homogenized in 1 ml of isolation buffer (0.32 M sucrose, 0.1 mM CaCl_2_ and 0.1 mM MgCl_2_, pH 7.4) at 4 °C in a 5 ml Potter-Elvehjem glass tube using a homogenizer stirrer HS-30E (Witeg Labortechnik GmbH, Wertheim, Germany) with 10 *strokes* at 700–900 rotations per min. The resulting homogenate was mixed with 6 ml sucrose 2 M and 2.5 mL CaCl_2_ 0.1 mM in an ultra-clear centrifuge tube (Beckman Coulter, Hospitalet de Llobregat, Spain). Then, 2.5 mL of sucrose 1 M containing 0.1 mM CaCl_2_ were slowly added on top of the tube to form a sucrose gradient. After centrifugation for 3 h at 100,000 x *g* at 4 °C, the synaptosomes were collected as the interphase between 1.25 M and 1 M sucrose. They were diluted 10 times in isolation buffer, centrifuged for 30 min at 15,000 × *g* at 4 °C and the resulting synaptosomal pellet was resuspended in 1 ml of isolation buffer for immediate use.

The separation of the presynaptic active zone, postsynaptic density and extrasynaptic fractions from striatal synapses was carried out as previously described^48^. Briefly, synaptosomes were diluted 1:10 in cold 0.1 mM CaCl_2_ and an equal volume of 2 x solubilization buffer (2% Triton X-100, 40 mM Tris, pH 6.0) was added to the suspension. The suspension was incubated for 30 min on ice with constant agitation and the insoluble material (synaptic junctions) pelleted (40,000 × *g* for 30 min at 4 °C). The supernatant (extrasynaptic fraction) was concentrated using an Amicon Ultra 15 10K (Merck Millipore) and proteins precipitated with six volumes acetone at −20 °C and recovered by centrifugation (18,000 × *g* for 30 min at −15 °C). The synaptic junctions pellet was washed in solubilization buffer (pH 6.0) and resuspended in 10 volumes of a second solubilization buffer (1% Triton X-100, 20 mM Tris at pH 8.0). After incubation for 30 min on ice with agitation, the mixture was centrifuged and the supernatant (presynaptic fraction) processed as described for the extrasynaptic fraction, whereas the insoluble pellet corresponds to the postsynaptic fraction. Protease inhibitors (Protease Inhibitor Cocktail Set III, Millipore, Temecula, CA, USA) were added to the suspension in all extraction steps. The protein concentration was determined by the BCA protein assay (Pierce Biotechnology) and 20 ug of each fraction, solubilized in 5% SDS, were added to SDS-PAGE sample buffer prior to freezing at −20 °C.

### cAMP assay in synaptosomes

Synaptosomal cAMP accumulation was measured using the LANCE Ultra cAMP kit (PerkinElmer, Waltham, MA, USA) as previously described^49^. In brief, total striatal synaptosomal membranes (0.5 μg) from GPR37+/+ and GPR37−/− mice were resuspended in stimulation buffer (HBSS 1X, 5 mM Hepes pH 7.4, 10 mM MgCl_2_, 0.1% BSA) and subsequently processed for cAMP accumulation. Thus, vehicle, forskolin (1 μM; Sigma-Aldrich) or CGS21680 (500 nM; Tocris Biosciences, Bristol, UK) were added for 30 min at 22 °C before the lysis and cAMP quantification in a POLARStar microplate reader (BMG Labtech, Durham, NC, USA). cAMP levels were calculated as previously described^49^.

### Striatal primary cell culture

Primary striatal neurons were cultured from GPR37+/+ and GPR37−/− mice embryos (E18). Briefly, after dissection, the striatum was treated with 1.25% trypsin for 10 min (Sigma-Aldrich) and mechanically dissociated with a flame polished Pasteur pipette. Neurons were plated onto poly-D-lysine (0.1 mg/ml) and laminin-coated (0.01 mg/ml) 6-wells plate (for cAMP accumulation assay) or 12-wells plate containing glass coverslips (for immunocytochemistry) in minimum essential medium (Invitrogen, Carlsbad, CA, USA) supplemented with 10% horse serum, 10% bovine serum, 1 mM pyruvic acid, and 0.59% glucose at a density of 80,000 cells/cm^2^. After 4–14 h, the medium was substituted with Neurobasal medium supplemented with penicillin (100 U/ml), streptomycin (100 μg/ml), 0.59% glucose, and B27 supplement (Invitrogen). Neurons were kept at 5% CO_2_, 37 °C and 95% humidity for 21 days *in vitro* (DIV) before the experiments.

### Immunofluorescence

For immunohistochemistry of brain slices, these were washed three times with PBS, permeabilized with 0.5% Triton X-100 in PBS for 2 hours and rinsed again three times with washing solution (0.05% Triton X-100 in PBS). The slices were then incubated with washing solution containing 10% normal donkey serum (NDS; Jackson ImmunoResearch Laboratories, Inc., West Grove, PA, USA) for 2 h at room temperature. Subsequently, slices were incubated with a new homemade rabbit anti-GPR37 polyclonal antibody, raised using a glutathione S-transferase (GST)-fusion protein containing amino acids 541–600 of GPR37 C terminus (anti-GPR37-C: 3 μg/ml), plus goat anti-A_2A_R (3 µg/ml; Frontier Institute Co. Ltd) in washing solution containing 10% NDS for 48h at 4 °C. Next, slices were washed with washing solution containing 1% NDS before the incubation with Cy3-conjugated donkey anti-rabbit IgG antibody (1/200; Jackson ImmunoResearch Laboratories) and Cy2-conjugated donkey anti-goat IgG antibody (1/200; Jackson ImmunoResearch Laboratories) in washing solution for 2 h at room temperature. Finally, slices were washed twice with 1% NDS in washing solution containing 10% NDS and then mounted with Vectashield immunofluorescence medium (Vector Laboratories, Peterborough, UK) in glass slides. Fluorescence striatal images were captured using a Leica TCS 4D confocal scanning laser microscope (Leica Lasertechnik GmbH).

For immunocytochemistry of DIV21 primary striatal neurons, these were grown on poly-D-lysine (0.1 mg/ml) and laminin-coated (0.01 mg/ml) coverslips, fixed in 4% paraformaldehyde for 15 min and washed with PBS containing 20 mM glycine (buffer A) to quench aldehyde groups. Neurons were then permeabilized with buffer A containing 0.2% Triton X-100 for 10 min. Subsequently, neurons were labeled for 1 h at 22 °C with a rabbit anti-GPR37-N antibody (1 μg/ml) and a goat anti-A_2A_R (1 μg/ml) washed, and stained with Cy5-conjugated donkey anti-rabbit IgG antibody (1/200) and Cy2-conjugated donkey anti-goat IgG antibody (1/200) (Jackson ImmunoReserach Laboratories Inc.). Coverslips were rinsed for 3 min in PBS, mounted with Vectashield immunofluorescence medium (Vector Laboratories) and examined using a Leica TCS 4D confocal scanning laser microscope (Leica Lasertechnik GmbH, Heidelberg, Germany).

For immunostaining of synaptosomes, total synaptosomes from striatum were fixed in 4% paraformaldehyde for 15 min using an end over end tube rotator at room temperature. Subsequently, 100 µl of a 0.125 mg/ml synaptosomal suspension was platted onto poly-D-lysine (0.1 mg/ml)-coated coverslips for 1 hour at 37 °C in a humidity chamber. Next, coverslips were processed as described for immunocytochemistry experiments with cultured neurons. Each coverslip was analysed by counting three different fields.

### Proximity ligation *in situ* assay

Duolink *in situ* PLA detection Kit (Olink Bioscience, Uppsala, Sweden) was performed in a similar manner as immunohistochemistry using rabbit anti-GPR37-C (3 μg/ml) plus goat anti-A_2A_R (3 µg/m) as a primary antibodies and the secondary antibodies following the manufacturer’s protocol, as previously described^19, 26^. Fluorescence images were acquired on a Leica TCS 4D confocal scanning laser microscope (Leica Lasertechnik GmbH) using a 60x N.A. = 1.42 oil objective from the selected area. High-resolution images were acquired as a z-stack with a 0.2 μm z-interval with a total thick of 5 μm. Nonspecific nuclear signal was eliminated from PLA images by substracting TO-PRO-3 Iodide (Thermo Fisher Scientific) labeling. Analyze particle function from Image J (NIH) was used to count particles larger than 0.3 μm^2^ for PLA signal and larger than 100 μm^2^ to discriminate neuronal from glia nuclei^50^. For each image several oligomer particles and neuron nuclei was obtained and ratio among them was calculated.

### Striatal primary cell culture cAMP assay

For cAMP assay, mouse striatal neurons were grown on a 6-wells plate. On DIV21, cAMP accumulation was measured using the cAMP HiRange assay Kit (CisBio, Bagnols-sur-Cèze, France). The growing media of striatal neurons was replaced by non-supplemented Neurobasal with adenosine deaminase (ADA, 5 µg/well; from Roche Diagnostics GmbH, Mannheim, Germany) and incubated for 2h. Subsequently, zardaverine (50 μM; Tocris Bioscience) was added and incubated for 30 min at 37 °C. Next, vehicle, forskolin (1 μM; Sigma-Aldrich) or CGS21680 (500 nM; Tocris Biosciences) were added for 30 min at 37 °C before the lysis with 250 µl of lysis buffer (CisBio). The neuronal extract was centrifuged at 13,000 × *g* for 30 min at 4 °C. The cAMP content in 10 µl of the supernatant was determined as previously described^49^.

### Catalepsy induction test

Catalepsy was induced in mice by the intracerebroventricular (i.c.v.) administration of CGS21680 (10 µg), as previously described^51^. The cataleptic response was measured as the duration of an abnormal upright posture in which the forepaws of the mouse were placed on a horizontal wooden bar (0.6 cm of diameter) that was located 4.5 cm above the floor. The latency to move at least one of the two forepaws was recorded 2h after CGS21680 administration.

### Open-field test

To evaluate the spontaneous locomotor activity, we performed the open field test. In brief, the mice were placed in the center of an activity field arena (30 × 30 cm, surrounded by four 50 cm high black painted walls) equipped with a camera above to record activity. Mice were administered (ip) with vehicle or SCH58261 (3.75 mg/kg; Abcam Biochemicals, Cambridge, UK) 15 min before measuring the exploratory behavior of the animals during a 10-min period. The total distance travelled and the activity within the outer and inner zone of the open field was analyzed using Spot tracker function from Image J (NIH). All behavioral tests were carried out in a sound attenuated room with 15 lux illumination. The apparatus and the objects were cleaned with a 70% alcohol solution and rinsed with water after each session.

### Statistics

The number of samples (n) in each set of experimental conditions is indicated in figure legends. Statistical analysis was performed by one-way ANOVA followed by Tukey *post-hoc* test, two-way ANOVA followed by Bonferroni *post-hoc* test or Student’s *t*-test when appropriate. Statistical significance was considered at *P* < 0.05.
